# Genetic and Environmental Determinants of Stress Responding

**DOI:** 10.35946/arcr.v34.4.12

**Published:** 2012

**Authors:** Toni-Kim Clarke, Charlotte Nymberg, Gunter Schumann

**Affiliations:** **Toni-Kim Clarke, Ph.D.,***is a postdoctoral fellow at the Center for Neurobiology and Behavior, Department of Psychiatry, University of Pennsylvania, Perelman School of Medicine, Philadelphia, Pennsylvania.*; **Charlotte Nymberg, M.Sc.,***is a doctural student, at the Medical Research Council (MRC) Social, Genetic, and Developmental Psychiatry Centre (SGDP), Institute of Psychiatry, King’s College, London, United Kingdom.*; **Gunter Schumann, M.D.,***is a professor and chair of biological psychiatry at the Medical Research Council (MRC) Social, Genetic, and Developmental Psychiatry Centre (SGDP), Institute of Psychiatry, King’s College, London, United Kingdom.*

**Keywords:** Alcohol dependence, alcoholism, alcohol use and abuse, alcohol and other drug use initiation, risk factors, genetic factors, environmental factors, stress, stress response, neurobiology, biological development, brain, hypothalamic–pituitary–adrenal axis, corticotropin-releasing factor system, animal studies, human studies, literature review

## Abstract

The risk for alcohol dependence throughout development is determined by both genetic and environmental factors. Genetic factors that are thought to modulate this risk act on neurobiological pathways regulating reward, impulsivity, and stress responses. For example, genetic variations in pathways using the brain signaling molecule (i.e., neurotransmitter) dopamine, which likely mediate alcohol’s rewarding effects, and in two hormonal systems involved in the stress response (i.e., the hypothalamic–pituitary–adrenal axis and the corticotropin-releasing factor system) affect alcoholism risk. This liability is modified further by exposure to environmental risk factors, such as environmental stress and alcohol use itself, and the effects of these factors may be enhanced in genetically vulnerable individuals. The transition from alcohol use to dependence is the result of complex interactions of genes, environment, and neurobiology, which fluctuate throughout development. Therefore, the relevant genetic and environmental risk factors may differ during the different stages of alcohol initiation, abuse, and dependence. The complex interaction of these factors is yet to be fully elucidated, and translational studies, ranging from animal studies to research in humans, and well-characterized longitudinal studies are necessary to further understand the development of alcohol dependence.

The development of alcohol dependence is a complex process influenced by both genetic and environmental risk factors (Prescott and Kendler 1999). The relative contributions of genetic and environmental influences fluctuate across development. During adolescence the initiation of alcohol use is strongly influenced by environmental factors (Dick et al. 2007; Heath et al. 1997; Karvonen 1995; Latendresse et al. 2008; McGue et al. 2000), whereas the genetic contribution to alcohol use at this stage is nonspecific and increases the risk for general externalizing behavior (Moffitt 1993; Moffitt et al. 2002). Specific genetic factors increasingly become relevant, however, as patterns of alcohol use are established (Hopfer et al. 2003; Pagan et al. 2006), particularly in mid-adulthood when dependence tends to emerge (Kendler et al. 2010; Schuckit et al. 1995). Gene–environment interactions also play a role because the influence of certain genetic factors seems to increase when a person is exposed to relevant environmental risk factors (Uhart and Wand 2009). Therefore, the development of dependence can be conceptualized within a temporal framework of genes, environment, and behavior.

The purpose of this review is to explore, within this framework, the contribution of some of the neurobiological systems that are important for the development of alcohol dependence. One of these is the mesolimbic dopaminergic system, which is involved in inducing the rewarding effects of alcohol and plays a central role in early alcohol use. Another pathway that also has been implicated in alcohol abuse, and particularly in the transition to alcohol dependence, involves two stress-response systems, the hypothalamic–pituitary–adrenal (HPA) axis and the extra-hypothalamic corticotropin-releasing factor (CRF) stress response system, which mediate the interaction of psychosocial stress and early alcohol use. Both of these systems exemplify how the effects of genes and environment may be augmented during critical periods of alcohol use and dependence across the lifespan. For example, the dopaminergic system undergoes developmental transformations during adolescence that are associated with increased reward sensitivity and risk taking (Spear 2000), which presents a window of vulnerability for exposure to alcohol and stress. Then, as alcohol use continues through life, chronic exposure to alcohol can enhance the activity of (i.e., upregulate) the HPA and CRF systems. This dysregulation of the stress response systems becomes a pathological feature of alcohol dependence, perpetuating chronic alcohol drinking based on an allostatic shift^1^ of the CRF system (Koob 2010). Moreover, the HPA, CRF, and dopaminergic systems can influence early alcohol drinking as a result of gene–environment interactions. This article will summarize the literature that has explored how genetic variation within the dopaminergic and stress response systems can influence the risk of alcohol dependence and how the exposure to relevant environmental risk factors and their interaction with genetic variants may influence alcoholism pathology. The effects of genes and environment on alcohol dependence will be discussed in a developmental framework from early childhood to adolescence as well as in the context of the development of dependence, when drinking behavior shifts from recreational use to dependence.

## Role of Dopaminergic and Stress Response Systems in Alcohol Initiation and Early Alcohol Use

### Environmental Factors and the Dopaminergic System

Several environmental factors have been shown to influence the initiation of alcohol consumption and its use during adolescence, including the level and quality of parental monitoring, peer-group influences, alcohol availability, and socioregional effects (Dick et al. 2007; Heath et al. 1997; Karvonen 1995; Latendresse et al. 2008; McGue et al. 2000). Thus, maternal and paternal alcohol use has been positively correlated with adolescent alcohol use at ages 14 and 17 (Latendresse et al. 2008). Moreover, the level of urbanization was found to correlate with alcohol use in Finnish adolescents at ages 16 and 18 (Karvonen 1995), and peer-group drinking behavior was one of the strongest predictors of problematic drinking in a cohort of Spanish adolescents (Ariza Cardenal and Nebot Adell 2000).

Once alcohol use has been initiated, neuronal networks are activated that engage the brain circuits mediating the rewarding effects of alcohol use (i.e., the reward neurocircuitry). This activation attributes salience to alcohol and serves as an incentive for alcohol use to continue (Robinson and Berridge 1993). Neuronal networks that are known to mediate these effects include those using the signaling molecules (i.e., neurotransmitters) glutamate and γ-aminobutyric acid (GABA) as well as the endogenous opioids (Gass and Olive 2008; Malcolm 2003; Oswald and Wand 2004). In addition, signal transmission involving the neurotransmitter dopamine in the mesolimbic system (Di Chiara and Imperato 1988) is particularly important for the establishment of regular alcohol consumption because alcohol-induced dopamine release is believed to contribute to the rewarding effects of alcohol (for reviews see, Soderpalm et al. 2009; Tupala and Tiihonen 2004). The mesolimbic system is a set of interconnected brain structures including the ventral tegmental area (VTA), nucleus accumbens (NAc), and components of the limbic system (e.g., the amygdala). Studies in rats found that alcohol consumption can increase dopamine signaling in the NAc (Weiss et al. 1996). Conversely, dopaminergic neurotransmission is decreased during withdrawal in the NAc and VTA of rats treated chronically with ethanol (Diana et al. 1993).

Environmental risk factors during early life and adolescence may interact with the dopaminergic system to influence alcohol intake. Two such factors are exposure to environmental stress and alcohol consumption itself. The developing adolescent brain undergoes substantial changes in the strength with which signals are transmitted between neurons (i.e., in synaptic plasticity) (Bava and Tapert 2010; Giedd 2003). These changes include increased dopaminergic inputs to the prefrontal cortex that peak during adolescence and decrease later in life (Kalsbeek et al. 1988; Rosenberg and Lewis 1994). Furthermore, dopamine levels in the NAc also peak during adolescence, before decreasing during subsequent brain maturation (Philpot and Kirstein 2004). These neuronal alterations are believed to promote sensation-seeking and risk-taking behavior during adolescence, which in turn increase the propensity for alcohol initiation and alcohol use (Spear 2000). Exposure to alcohol and/or stress during early life (i.e., from the prenatal period through adolescence) has been shown to have lasting consequences on the dopamine system that have a significant impact on the risk for alcohol abuse.

### The Effects of Early Alcohol Use on the Dopaminergic System

Studies in rats found that exposure to alcohol during the prenatal period decreases the levels of two important enzymes involved in regulating dopamine activity—the dopamine transporter and the dopamine hydroxylase enzyme—in the VTA (Szot et al. 1999). Moreover, rats chronically treated with ethanol during adolescence displayed persistently elevated baseline dopamine levels in the NAc during adulthood, even after a period of 15 days abstinence (Badanich et al. 2007). Finally, repeated ethanol injections in preadolescent and adolescent rats increased subsequent dopamine activity in the NAc, with the largest increases observed in preadolescence. Early ethanol exposure in these rats decreased the ability of subsequent ethanol injections to elicit dopamine release from the NAc (Philpot and Kirstein 2004). These findings suggest that ethanol exposure in early life may influence the response to alcohol in later life. Indeed, additional studies have confirmed that both pre- and postnatal exposure to alcohol increase the sensitivity of rats to the locomotor effects of alcohol and to an agent that mimics dopamine’s effects (i.e., a dopamine agonist), apomorphine (Barbier et al. 2009). Therefore, at least in rodents, early alcohol exposure seems to confer lasting effects on neuronal dopamine activity that can alter behavioral responses to subsequent alcohol exposure. Indeed, rats chronically treated with ethanol both prenatally and during adolescence also show an increased preference for alcohol and increased alcohol intake as adults (Barbier et al. 2009; Pascual et al. 2009). Furthermore, stress-induced alcohol consumption was associated with an earlier age of drinking onset in Wistar rats (Fullgrabe et al. 2007; Siegmund et al. 2005).

Studies in humans have confirmed the potential long-lasting impact of early alcohol exposure, demonstrating that an early initiation of alcohol use is associated with an increased risk of later problems with alcohol. For example, Hawkins and colleagues (1997) noted that the earlier drinking is initiated in adolescence, the greater the levels of alcohol misuse at ages 17 to 18. Furthermore, people who begin drinking at age 14 or younger are more likely to become alcohol dependent later in life (Grant and Dawson 1997). Few studies have been conducted to determine the precise mechanism by which early alcohol exposure affects the risk for subsequent alcohol abuse and dependence. However, Pascual and colleagues (2009) demonstrated that in adolescent rats chronically treated with ethanol, two neurotransmitter receptors—dopamine receptor 2 (DRD2) and glutamate receptor (NMDAR2B)—show lower levels of a chemical modification (i.e., phosphorylation) in the prefrontal cortex compared with adults chronically treated with ethanol. This finding suggests that alcohol use during adolescence causes neurobiological changes to the dopamine system that are not observed in adult animals.

**The Extrahypothalamic Corticotropin-Releasing Factor System and the Transition to Alcohol Dependence**

As described in the main article, corticotropin-releasing factor (CRF) is a key component of one of the body’s main stress response systems, the hypothalamic–pituitary–adrenal (HPA) axis. Moreover, activation of the HPA axis in response to stressful situations as well as alcohol ingestion plays an important role in the development of alcohol dependence. However, studies in rodents and macaques have shown that enhanced activity (i.e., upregulation) of the CRF system in response to chronic alcohol exposure in several brain regions not immediately related to the HPA system (e.g., the amygdala) also is a key characteristic of alcohol dependence. CRF is an anxiety-inducing peptide, and rodent models of motivation have demonstrated that CRF, administered either directly into the brain or under the skin, induces conditioned place aversion (Cador et al. 1992). In addition, studies in mice found that transient elevation of CRF levels in the fore-brain during early development increased anxiety in later life compared with control animals (Kolber et al. 2010).

Studies of a rat strain bred for high alcohol preference (i.e., the mSP rats) found that the animals display an increased behavioral sensitivity to stress and a lowered threshold for stress-induced reinstatement of alcohol-seeking behavior (Hansson et al. 2006). Gene expression analyses across different brain regions of the mSP strain revealed a significantly enhanced expression of a gene, *CRF1,* which encodes one of the CRF receptors. Additional gene sequence analyses of the mSP rats identified a DNA variation (i.e., polymorphism) in a regulatory region (i.e., the promoter) of the *CRF1* gene that is unique to the mSP rats, suggesting that segregation of this polymorphism may have occurred during selection for the alcohol preference trait. However, alcohol consumption reduced CRF1 levels in the amygdala and the nucleus accumbens (NAc) in mSP rats, indicating that the animals may consume alcohol to reduce CRF activity in these regions (Hansson et al. 2007).

Studies in *Rhesus* macaques also have confirmed the link between the CRF system, stress, and alcohol because a polymorphism (–248C/T) in the promoter of the CRF gene was associated with differential behavioral and hormonal responses to stress. Animals that carried the T allele DNA variant at this site displayed greater HPA axis responses to separation stress and increased alcohol intake if they were exposed to early-life adversity in the form of peer rearing (Barr et al. 2009). These findings demonstrate that genetic variation in the CRF system associated with increased sensitivity to stressors also is correlated with increased alcohol consumption in both rats and primates. Because alcohol consumption is known to reduce the activity of the HPA axis, hyperactivity of this system in animals carrying risk variants of the CRF gene likely is a motivating factor for alcohol consumption in these animals, and this effect is enhanced when the animals are exposed to stressors.

Animal studies also have demonstrated that agents that block the activity of the CRF1 receptor (i.e., CRF1 antagonists) may be suitable for treatment of alcohol dependence (Gehlert et al. 2007). Although animals do not exhibit all aspects of alcohol dependence found in humans, certain components of the disorder can be modeled in rodents. Thus, researchers induced a “postdependent state” in rats by first subjecting the animals to involuntary intermittent exposure to alcohol vapor and then allowing them 3 weeks of recovery from the exposure (Sommer et al. 2008). After this recovery period, the animals displayed increased CRF1 levels in the amygdala, comparable to those observed in mSP rats at baseline. In addition, the postdependent animals exhibited increased fear suppression of behavior that persisted for 3 months after cessation of alcohol exposure, as well as increased voluntary alcohol consumption. This postdependent phenotype could be reversed by a CRF1 antagonist, 3-(4-chloro-2-morpholin-4-yl-thiazol-5-yl)-8-(1-ethylpropyl)-2,6-dimethyl-imidazo[1,2-b]pyridazine (MTIP) (Funk et al. 2006; Sommer et al. 2008), confirming the role of increased CRF activity during alcohol dependence. Other studies also demonstrated that selective CRF1 antagonists reduced alcohol self-administration in alcohol-dependent animals but had no effect in alcohol-naïve animals (Funk et al. 2006, 2007). The exposure to stress, which often triggers relapse in abstaining alcoholics, also reinstates alcohol-seeking behavior in postdependent animals. CRF1 antagonists can suppress this behavior in animals (Le et al. 2000; Liu and Weiss 2002; Marinelli et al. 2007), further confirming their relevance as a potential pharmacotherapy for alcohol dependence. Finally, CRF1 antagonists can block the anxiety-like responses exhibited during withdrawal from alcohol in animals (Breese et al. 2005).

The potential of CRF1 antagonists in the treatment of alcohol dependence now also is being considered in humans. CRF1 antagonists previously have been assessed in the treatment of depression and anxiety (Zobel et al. 2000) and Phase II/Phase III clinical trials with these agents currently are underway for the treatment of alcohol use disorders (www.clinicaltrials.gov; Zorrilla and Koob 2010). The results of these trials may pave the way for the clinical consideration of CRF1 antagonists for addictive disorders. If such compounds are efficacious in humans, pharmacogenetic studies may identify those patients who are most amenable to CRF1 antagonist treatment, especially among those who are exposed to high levels of lifetime stress.

ReferencesBarrCSDvoskinRLGupteMFunctional CRH variation increases stress-induced alcohol consumption in primatesProceedings of the National Academy of Sciences of the United States of America106145931459820091970654610.1073/pnas.0902863106PMC2732884BreeseGRChuKDayasCVStress enhancement of craving during sobriety: A risk for relapseAlcoholism: Clinical and Experimental Research2918519520051571404210.1097/01.alc.0000153544.83656.3cPMC2868509CadorMAhmedSHKoobGFCorticotropin-releasing factor induces a place aversion independent of its neuroendocrine roleBrain Research5973043091992147300110.1016/0006-8993(92)91487-yFunkCKO'DellLECrawfordEFKoobGFCorticotropin-releasing factor within the central nucleus of the amygdala mediates enhanced ethanol self-administration in withdrawn, ethanol-dependent ratsJournal of Neuroscience26113241133220061707966010.1523/JNEUROSCI.3096-06.2006PMC6674550FunkCKZorrillaEPLeeMJCorticotropin-releasing factor 1 antagonists selectively reduce ethanol self-administration in ethanol-dependent ratsBiological Psychiatry61788620071687613410.1016/j.biopsych.2006.03.063PMC2741496GehlertDRCippitelliAThorsellA3-(4-Chloro-2-morpholin-4-yl-thiazol-5-yl)-8-(1-ethyl-propyl)-2,6-dimethyl- imidazo[1,2-b]pyridazine: A novel brain-penetrant, orally available corticotropin-releasing factor receptor 1 antagonist with efficacy in animal models of alcoholismJournal of Neuroscience272718272620071734440910.1523/JNEUROSCI.4985-06.2007PMC6672492HanssonACCippitelliASommerWHRegion-specific down-regulation of Crhr1 gene expression in alcohol-preferring msP rats following ad lib access to alcoholAddiction Biology12303420071740749510.1111/j.1369-1600.2007.00050.xHanssonACCippitelliASommerWHVariation at the rat Crhr1 locus and sensitivity to relapse into alcohol seeking induced by environmental stressProceedings of the National Academy of Sciences of the United States of America103152361524120061701582510.1073/pnas.0604419103PMC1622806KolberBJBoyleMPWieczorekLTransient early-life forebrain corticotropin-releasing hormone elevation causes long-lasting anxiogenic and despair-like changes in miceJournal of Neuroscience302571258120102016434210.1523/JNEUROSCI.4470-09.2010PMC2969849LeADHardingSJuzytschWThe role of corticotrophin-releasing factor in stress-induced relapse to alcohol-seeking behavior in ratsPsychopharmacology (Berlin)15031732420001092376010.1007/s002130000411LiuXWeissFAdditive effect of stress and drug cues on reinstatement of ethanol seeking: Exacerbation by history of dependence and role of concurrent activation of corticotropin-releasing factor and opioid mechanismsJournal of Neuroscience227856786120021222353810.1523/JNEUROSCI.22-18-07856.2002PMC6758095MarinelliPWFunkDJuzytschWThe CRF1 receptor antagonist antalarmin attenuates yohimbine-induced increases in operant alcohol self-administration and reinstatement of alcohol seeking in ratsPsychopharmacology (Berlin)19534535520071770506110.1007/s00213-007-0905-xSommerWHRimondiniRHanssonACUpregulation of voluntary alcohol intake, behavioral sensitivity to stress, and amygdala Crhr1 expression following a history of dependenceBiological Psychiatry6313914520081758588610.1016/j.biopsych.2007.01.010ZobelAWNickelTKunzelHEEffects of the high-affinity corticotropin-releasing hormone receptor 1 antagonist R121919 in major depression: The first 20 patients treatedJournal of Psychiatric Research3417118120001086711110.1016/s0022-3956(00)00016-9ZorrillaEPKoobGFProgress in corticotropin-releasing factor-1 antagonist developmentDrug Discovery Today1537138320102020628710.1016/j.drudis.2010.02.011PMC2864802

### The Effects of Environmental Stress on the Dopaminergic System

Environmental stress is one of the most pertinent risk factors for alcohol dependence. The exposure to early-life stress sensitizes animals to drugs of abuse (Fahlke et al. 1994; Piazza et al. 1991; Shaham and Stewart 1994) and also increases alcohol consumption in later life (Fahlke et al. 2000). Alterations in the dopaminergic mesolimbic system that persist into adulthood are believed to explain, at least in part, these behavioral adaptations (for review, see Rodrigues et al. 2011). For example, studies in rats found that chronic exposure to cold stress in adolescence altered both basal and stress-evoked release of dopamine and another neurotransmitter, norepinephrine,^2^ in the medial prefrontal cortex, NAc, and striatum compared with stress-naïve rats (Gresch et al. 1994). Other studies in Sprague-Dawley rats demonstrated that stress caused by separation from the mother during the first 2 weeks of life blunted the animals’ dopamine response to restraint stress in adulthood (Jahng et al. 2010). Although no human studies analyzing the effect of early-life stress and alcohol sensitization exist, imaging studies using functional magnetic resonance imaging (fMRI) to analyze reward anticipation have found that childhood adversity is associated with blunted subjective responses to reward-predicting cues as well as with impaired reward-related learning and motivation (Dillon et al. 2009). Such findings demonstrate that early environmental experiences can alter the impact of a reward and that similar effects can be observed across species.

Other studies have evaluated the effects of early-life stress on alcohol consumption or alcohol dependence. Such studies found that even exposure to prenatal stress can have an impact on later alcohol-related behaviors because the offspring of mice that repeatedly were restrained during the last 7 days of gestation subsequently demonstrated enhanced alcohol consumption—an effect that has been linked to persistently elevated dopaminergic and glutamatergic neurotransmission in the forebrain (Campbell et al. 2009). In humans, retrospective studies examining early-life experiences and alcohol consumption found that childhood stressors were associated with alcohol dependence during adulthood (Ducci et al. 2009; Pilowsky et al. 2009). In a study of the adult American population (i.e., the National Epidemiologic Survey on Alcohol and Related Conditions [NESARC]), two or more stressful life events in childhood significantly increased the risk for alcohol dependence in adulthood (Pilowsky et al. 2009). Furthermore, early initiation of alcohol use in human adolescents is associated with exposure to traumatic life events and symptoms of posttraumatic stress disorder (Wu et al. 2010).

Thus, exposure to stress and/or alcohol consumption during early life may influence dopaminergic neurotransmission, with lasting adaptations into adulthood and notable consequences for subsequent alcohol use. However, the impact on different individuals varies, and a portion of this variability can be attributed to genetic factors. Indeed, studies of rats have shown that exposure to chronic unpredictable stress increases the levels of a dopamine-metabolizing enzyme, tyrosine hydroxylase (TH), in the VTA but that the extent of this increase differs drastically between different rat strains (Ortiz et al. 1996). Additional research in *Rhesus macaques* identified a variation (i.e., polymorphism) in the gene encoding dopamine receptor 1 (DRD1)^3^ that was associated with increased alcohol consumption in animals exposed to peer-rearing conditions compared with maternally reared animals that carried the same polymorphism (Newman et al. 2009).

Studies in humans also have shown that genetic factors mediate the effects of stress and alcohol on the risk for alcohol dependence. Schmid and colleagues (2009) analyzed 291 young adults in the Mannheim Study of Children at Risk for two polymorphisms in the gene encoding the dopamine transporter. The investigators found that the age of first alcohol use and of intensive alcohol consumption mediated the association between these polymorphisms and early alcohol abuse and dependence. Genetic variation in another gene, *KCNJ6*, which is expressed in the brain, mediates the effects of early-life stress on alcohol abuse in adolescence. It induces inhibition of neuronal signaling at the level of the signal-receiving (i.e., postsynaptic) dopaminergic neurons (Kuzhikandathil et al. 1998). Furthermore, the protein encoded by the *KCNJ6* gene, the membrane potasium channel GIRK2, is co-expressed in TH-positive cells of mice (Schein et al. 1998). Individuals who carry a certain *KCNJ6* variant and are exposed to high levels of psychosocial stress in early life display increased risky drinking behavior in adolescence; moreover, the same polymorphism is associated with alcohol dependence in adults (Clarke et al. 2011).

Genes in other neurobiological systems also mediate the effects of early-life stress on alcohol consumption, including genes encoding the serotonin receptor (Laucht et al. 2009) and the GABA receptor subunit α-2 (*GABRA2*) (Enoch et al. 2010). Another important gene is that encoding the μ-opioid receptor (*OPRM1)*. It also moderates the effects of stress and alcohol with implications not only for alcohol use but also for recovery from alcohol dependence. Alcohol activates the μ-opioid receptor in the VTA, which causes inhibition of GABAergic neurons; this in turn results in disinhibition of dopaminergic neurons and, thus, increased dopamine release in the ventral striatum (Spanagel 2009). In macaques, a certain polymorphism in the *OPRM1* gene (i.e., the C77G polymorphism) predicts the degree of distress upon exposure to maternal separation (Barr et al. 2008). In humans, the equivalent polymorphism (i.e., the A118G polymorphism) is associated with the quality of parent–child interactions under conditions of poor parenting (Copeland et al. 2011). Finally, in both macaques and humans the same polymorphisms are associated with subjective/behavioral responses to alcohol (Barr et al. 2007, 2008; Ramchandani et al. 2010). The role of this polymorphism further has been demonstrated in studies using a μ-opioid receptor antagonist, naltrexone, that commonly is used to treat alcohol dependence. In heavy drinkers, the A118G polymorphism mediates the effects of naltrexone on positive mood, craving, and enjoyment from alcohol (Ray and Hutchison 2004). Furthermore, the presence or absence of the A118G polymorphism can help predict which individuals will benefit from naltrexone treatment for alcohol dependence (Oslin et al. 2003).

Taken together, the findings described here indicate that early exposure to alcohol and stress can increase the subsequent risk for alcohol dependence, at least in part because they induce changes in the dopamine system. However, these effects are moderated by genetic factors in the dopamine pathways and other neurobiological systems.

## Brain Stress Response Systems and the Development of Alcohol Dependence

As indicated by the observations discussed in the preceding section, the dopamine system is an important neurobiological system mediating early alcohol use. In addition, stress response systems in the brain have been implicated in alcohol initiation and in the escalation of alcohol use from episodic use to abuse and, ultimately, dependence. Stress responses are crucial for survival by allowing the organism to coordinate appropriate behavioral adaptations to adverse stimuli and are essential homeostatic processes. Central components of the stress response include activation of the HPA axis, increases in norepinephrine turnover in a brain region, the locus coeruleus, and activation of CRF systems (Habib et al. 2001). CRF acts through two pathways. First, it acts as a signaling hormone inside the HPA axis, where it is released from the paraventricular nucleus of the hypothalamus. It then is transported to the anterior pituitary, where it binds to CRF receptors (CRF1 and CRF2), thereby eliciting the release of adreno-corticotrophic hormone (ACTH). ACTH production ultimately results in the release of stress hormones (i.e., glucocorticoids) from the adrenal glands. The main glucocorticoid in humans is cortisol. Second, CRF acts outside of the hypothalamus (i.e., extrahypothalamically) because immunological tests have detected its presence in the extended amygdala and the brainstem (Swanson et al. 1983).

Studies have demonstrated that exaggerated HPA axis responses to stress can precede the onset of alcoholism. Nondependent sons of alcoholic fathers (who are at increased risk of alcoholism) displayed increased cortisol and ACTH responses to psychosocial stress compared with people with no family history of alcoholism (Uhart et al. 2006; Zimmermann et al. 2004*a*, *b*). Furthermore, alcohol had a greater attenuating effect on ACTH and a related hormone (i.e., arginine vasopressin [AVP]) in people with alcoholic fathers, suggesting that alcohol may be more rewarding for such individuals (Zimmermann et al. 2004*b*). These findings also indicate that interindividual differences in HPA axis activity may underlie some of the variation observed in the vulnerability to alcohol dependence.

As alcohol dependence develops, the stress response systems are upregulated, and this hyperactivity may in fact be a pathological component of dependence (Koob 2008). It has been hypothesized that as dependence develops, the motivation for alcohol use shifts from positive reinforcement, whereby alcohol is consumed for its pleasurable effects, to negative reinforcement—that is, the drinker consumes alcohol to alleviate the negative emotional effects encountered during withdrawal and into protracted abstinence (Koob and Le Moal 2008). The development of negative emotional states has been proposed to include the recruitment and subsequent deregulation of various brain stress system, including the HPA axis, extrahypothalamic CRF, and various others^4^ (George et al. 2008; Koob 2008). Genetic variation in genes encoding components of these stress response systems therefore may be relevant for the risk for alcohol dependence.

### Genetic Influences on Stress Responding and Their Role in Alcohol Dependence

The variability between individuals in stress responding results at least partially from inherited factors (Armbruster et al. 2009; Linkowski et al. 1993; Meikle et al. 1988) that also may influence the risk of alcohol dependence. For example, polymorphisms that affect only a single DNA building block (i.e., single nucleotide polymorphisms [SNPs]) in the gene encoding CRF1 were associated with alcohol consumption and a lifetime prevalence of drunkenness in two independent samples (Treutlein et al. 2006). One of those polymorphisms, known as rs1876831, was found to moderate the effects of stress on drinking. Thus, adolescents at age 15 who had experienced negative life events in the past 3 years and who carried the variant (i.e., allele) of rs1876831 that was associated with increased risk of drinking displayed increased alcohol consumption per drinking occasion and greater lifetime rates of heavy drinking (Blomeyer et al. 2008). A similar effect also was observed at age 19, when the risk allele was associated with earlier age of onset of alcohol use and higher alcohol consumption in individuals exposed to stressful life events (Schmid et al. 2010). Furthermore, a gene–environment interaction was detected with a combination of several gene variants (i.e., a haplotype) in the *CRF1* gene (which also contains rs1876831) and childhood sexual abuse in a large cohort of Australians recruited for the Nicotine Genetics Project (Saccone et al. 2007). Individuals who had experienced childhood abuse but carried a protective polymorphism of the *CRF1* gene had lower lifetime alcohol consumption scores and rates of alcohol dependence (Nelson et al. 2009).

Further genetic factors mediating the association between the stress response and alcohol consumption are found in genes encoding the receptors to which cortisol binds after it is released from the adrenal gland when the HPA becomes activated (Bjorntorp 2001). Cortisol binds to glucocorticoid receptors (GRs) that are made up of two identical subunits (i.e., form homodimers). These receptors interact with certain DNA sequences, glucocorticoid response elements (GREs), in the target genes, thereby activating those genes as part of the stress response (Gower 1993; Simons et al. 1992). The GRs are encoded by a family of genes known as nuclear member subfamily 3 (*NR3C*) genes.

Researchers have identified functional polymorphisms in the genes encoding two receptors, NR3C1 and NR3C2, which are associated with differential responses to stress (Wust et al. 2004). For example, a SNP, N363S that results in an altered receptor, protein (i.e., a non-synonymous SNP) in *NR3C1* is associated with increased glucocorticoid sensitivity (Huizenga et al. 1998) as well as elevated levels of cortisol in the saliva of healthy people in response to psychosocial stress (Wust et al. 2004). Moreover, a haplotype that includes three SNPs and is located in a noncoding region of the *NR3C1* gene also is associated with enhanced sensitivity to glucocorticoids (Stevens et al. 2004). Because chronic alcohol consumption can increase HPA axis activity in animals and humans (Rivier 1996; Rivier and Lee 1996; Waltman et al. 1994), polymorphisms in genes encoding components of the HPA axis may increase the risk for alcohol abuse. Indeed, a recent study of 26 SNPs across the *NR3C1* gene in 4,534 adolescents identified several variants that were associated with onset of drinking and drunkenness by age 14, suggesting that genetic variation in *NR3C1* can influence the risk of alcohol abuse in adolescence (Desrivieres 2010). Likewise, variants in the gene encoding the ACTH precursor, promelanocortin *(POMC)*, have been associated with substance abuse, including alcohol abuse (Zhang et al. 2009).

Genes encoding components of the norepinephrine stress response system also have been linked to variability in the response to stress. Thus, polymorphisms in the *ADRA2A* gene, which encodes adrenergic receptors that inhibit norepinephrine release from the neuron, are associated with certain aspects of the stress response as determined by measuring blood pressure and heart rate (Finley et al. 2004). In addition, variants in the *ADRA2A* gene are associated with alcohol abuse phenotypes in humans. For example, in a study analyzing 23 SNPs in *ADRA2A* as well as in a gene *SLC6A2* (which encodes the norepinephrine transporter, NET1) in association with adult alcohol dependence identified two SNPs in *ADRA2A* associated with a positive family history of alcoholism and four SNPs in *SLC6A2* associated with adult alcohol dependence (Clarke et al. 2010).

All of these studies demonstrate that genes that regulate stress responding also influence the risk for alcohol dependence. Thus, people who display increased sensitivity to stress may consume alcohol to dampen the exaggerated stress responses and therefore may find alcohol more rewarding. These people also may more readily experience the negative emotional states associated with withdrawal after chronic alcohol exposure, which may accelerate the transition to dependence. However, the precise relationship between genes, stress, and alcohol use is complex, and gene–environment interactions are notoriously difficult to elucidate (Flint and Munafo 2008). Therefore, translational studies analyzing the effects of genetic factors and stress and their interactions under tightly controlled experimental conditions using animal models are warranted (Barr and Goldman 2006). Indeed, the study of the extrahypothalamic CRF system in animals has helped to clearly delineate the role of brain stress systems in the pathology of alcoholism, and this system is now a plausible target for future alcoholism pharmacotherapies. (For more information on these studies, see the sidebar “The Extrahypothalamic CRF System and the Transition to Alcohol Dependence.”)

Another confounding issue for the study of gene–environment interactions is that many studies are conducted retrospectively, and the participants’ recall of environmental risk factors may not be accurate. Therefore, prospective longitudinal studies are of great importance to advance the field of gene–environment interactions in alcohol dependence. One study that illustrates how such methodological issues can be addressed is the IMAGEN study, a longitudinal initiative funded by the Framework 6 program of the European Commission and the Medical Research Council that tracks the interplay between genetic polymorphisms and environmental stressors from early adolescence onward. The study collects neuropsychological, behavioral, and functional/structural neuroimaging data and also conducts genetic analyses on a sample of 2,000 adolescents from age 14 onward. (For more information on this study, see the sidebar “The IMAGEN Study.”)

**The IMAGEN Study**

The IMAGEN study (www.imagen-europe.com) is the first study aimed at identifying the genetic and neurobiological basis of individual variability in impulsivity, reinforcer sensitivity, and emotional reactivity, as well as determining their predictive value for the development of common psychiatric disorders. The data collection of IMAGEN began in 2007. Since then, the study has collected comprehensive behavioral and neuropsychological data, as well as functional/structural neuroimaging data for 2,000 14-year-old adolescents. These data are complemented by genome-wide association (GWA) data on the study participants. These genetic analyses target approximately 600,000 DNA markers distributed across the genome, using the Illumina Quad 660 chip.

Data from the first wave of IMAGEN became available in 2010 in an extensive database (Schumann et al. 2010), and since then several articles have been published on the dataset, contributing toward a greater understanding of the adolescent brain. For example, Peters and colleagues (2010) showed that adolescent smokers display lower activation of the ventral striatum during reward anticipation compared to their nonsmoking peers. Other studies identified gender-dependent amygdala lateralization during face processing and created probabilistic maps of the face network in the adolescent brain (Schneider et al. 2010; Tahmasebi et al. 2010).

The sample will be followed up at age 16 to investigate the predictive value of genetic factors and intermediate phenotypes for the development of mental disorders, such as alcohol dependence. The full dataset from the follow-up will be completed in 2012. A second follow-up is planned to be completed when the participants reach age 18.

In conclusion, IMAGEN integrates technological and methodological advances in the field of cognitive neuroscience as well as in the fields of human and molecular genetics. This comprehensive approach, together with the large sample sizes, will provide new insights into the interplay between genes and environments that results in individual variability in brain structure, function, and psychological traits. The complex phenotypic and genotypic profiling provided by IMAGEN will be vital in identifying biomarkers that aid in earlier diagnosis and in the developments of treatments for psychiatric disorders, including alcohol dependence.

ReferencesSchneiderSPetersJBrombergUBoys do it the right way: Sex-dependent amygdala lateralization during face processing in adolescentsNeuroImage561847185320112131646710.1016/j.neuroimage.2011.02.019SchumannGLothEBanaschewskiTThe IMAGEN study: Reinforcement-related behaviour in normal brain function and psychopathologyMolecular Psychiatry151128113920102110243110.1038/mp.2010.4TahmasebiAMArtigesEBanaschewskiTCreating probabilistic maps of the face network in the adolescent brain: A multicentre functional MRI studyHuman Brain Mapping2010[Epub ahead of print]2141656310.1002/hbm.21261PMC6869899

## Conclusion and Future Perspectives

Dopaminergic and stress response pathways jointly are engaged upon the commencement of alcohol consumption. Genetic polymorphisms within these pathways may affect the risk of developing alcohol dependence. The effects of exposure to environmental stressors that increase the risk of developing alcohol dependence may be augmented in genetically vulnerable individuals. In some cases, these genetic variants may vary the impact that a particular stressor has within a specific time window (see the figure). To elucidate the role of alcohol usage as a consequence of environmental stressors, and as an environmental stressor in itself, longitudinal studies of the interplay between genes and environments are needed.

The IMAGEN study is an ongoing longitudinal study that attempts to address the role of genes and the environment in alcohol use. The extensive phenotypic database available from this study will allow researchers to test the hypothesis that overactivity of the brain’s stress systems, resulting from childhood maltreatment and neglect, may affect brain development and ultimately behaviors such as alcohol use. Alcohol use patterns of the IMAGEN participants are recorded to investigate the long-term effects of early intoxication on cognitive development and behavior. Finally, genetic analyses investigating the association of genetic markers distributed across the genome with specific traits or behaviors (i.e., genomewide association data) are available for each participant and may demonstrate the relationship between genes of the stress response system and intermediate phenotypes (Schumann et al. 2010).

Longitudinal gene–neuroimaging studies, such as the IMAGEN study, aim to clarify the role of the HPA axis and supplementary stress systems in the development and maintenance of alcohol dependence. Such studies will elucidate how alcohol use fluctuates throughout development under the influence of genetic and environmental factors. A better understanding of these factors will promote novel therapies for alcohol dependence as well as approaches to prevent the disorder.

## Figures and Tables

**Figure:**
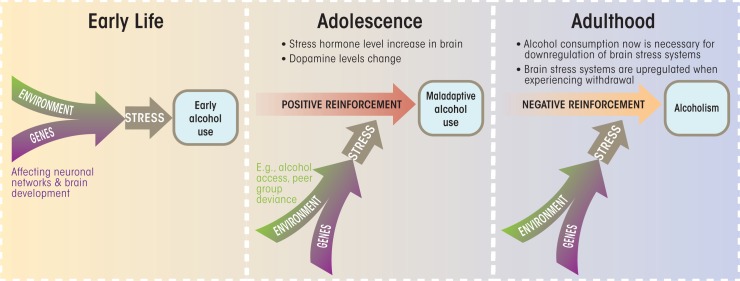
Schematic depiction of the typical progression from alcohol use to alcohol dependence. Both genetic and environmental factors influence each stage of disease progression. Early-life experiences, including prenatal environments and early-life stressors, may affect the onset of alcohol use. In adolescence, heightened sensation seeking, resulting from an increase in cortical dopamine neurons, often results in experimentation with alcohol. In adulthood, alcohol use may occur to downregulate brain stress systems in individuals suffering from alcohol dependence. Thus, early alcohol use is motivated by positive reinforcement, whereas later stages are driven by negative reinforcement, when alcohol is consumed to alleviate negative emotional states.

## Footnotes

1 The term allostasis refers to the process through which various biological processes attempt to restore the body’s internal balance (i.e., homeostasis) when an organism is threatened by various types of stress in the internal or external environment. Allostatic responses can involve alterations in HPA axis function, the nervous system, various signaling molecules in the body, or other systems.
